# The Retrograde Connections and Anatomical Segregation of the Göttingen Minipig Nucleus Accumbens

**DOI:** 10.3389/fnana.2016.00117

**Published:** 2016-12-05

**Authors:** Anders C. Meidahl, Dariusz Orlowski, Jens C. H. Sørensen, Carsten R. Bjarkam

**Affiliations:** ^1^Department of Neurosurgery, Department of Clinical Medicine, Faculty of Health, Center for Experimental Neuroscience, Aarhus University Hospital, Aarhus UniversityAarhus, Denmark; ^2^Department of Neurosurgery, Institute of Clinical Medicine, Aalborg University HospitalAalborg, Denmark

**Keywords:** fluorogold, striatum, deep brain stimulation, treatment resistant depression, sus scrofa, calbindin, OCD

## Abstract

Nucleus accumbens (NAcc) has been implicated in several psychiatric disorders such as treatment resistant depression (TRD), and obsessive-compulsive disorder (OCD), and has been an ongoing experimental target for deep brain stimulation (DBS) in both rats and humans. In order to translate basic scientific results from rodents to the human setting a large animal model is needed to thoroughly study the effect of such therapeutic interventions. The aim of the study was, accordingly, to describe the basic anatomy of the Göttingen minipig NAcc and its retrograde connections. Tracing was carried out by MRI-guided stereotactic unilateral fluorogold injections in the NAcc of Göttingen minipigs. After 2 weeks the brains were sectioned and subsequently stained with Nissl-, autometallographic (AMG) development of myelin, and DARPP-32 and calbindin immunohistochemistry. The minipig NAcc was divided in a central core and an outer medial, ventral and lateral shell. We confirmed the NAcc to be a large and well-segregated structure toward its medial, ventral and lateral borders. The fluorogold tracing revealed inputs to NAcc from the medial parts of the prefrontal cortex, BA 25 (subgenual cortex), insula bilaterally, amygdala, the CA1-region of hippocampus, entorhinal cortex, subiculum, paraventricular and anterior parts of thalamus, dorsomedial parts of hypothalamus, substantia nigra, ventral tegmental area (VTA), the retrorubral field and the dorsal and median raphe nuclei. In conclusion the Göttingen minipig NAcc is a large ventral striatal structure that can be divided into a core and shell with prominent afferent connections from several subrhinal and infra-/prelimbic brain areas.

## Introduction

Nucleus accumbens (NAcc) has been implicated in several psychiatric disorders such as depression and obsessive compulsive disorder (OCD) and has accordingly been an ongoing experimental target for deep brain stimulation (DBS) in rats, primates and humans (Schlaepfer et al., 2007; Bewernick et al., 2010; de Koning et al., 2011; Li et al., 2013).

In humans NAcc forms the main part of the so-called ventral striatum located beneath the anterior limb of the internal capsule where the head of the caudate nucleus and the ventro-anterior part of putamen meet (Lucas-Neto et al., 2013). NAcc borders medially the lateral septal nuclei and ventrally the olfactory tubercle (Basar et al., 2010). The dorsal and lateral borders of NAcc to the rest of striatum are, however, more difficult to define. In fact cytoarchitecture and immunohistochemical characteristics indicate that no sharp borders between NAcc and the rest of the striatum exist (Voorn et al., 2004) and the NAcc blends in with the rest of the striatum in Nissl stained sections (Heimer et al., 1997).

NAcc was initially divided into two regions—an outer medial, ventral, and lateral shell and a more dorsal and centrally located core based on differences in cytology and chemoarchitectonics as well as a differential distribution of cholecystokinin-immunoreactivity (Záborszky et al., 1985). Today calbindin immunohistochemistry is generally considered the most accepted staining for making this distinction (Meredith et al., 1996; Heimer et al., 1997; Groenewegen et al., 1999; Brauer et al., 2000).

Previous tracing studies of NAcc connectivity, primarily in rats, show afferent connections from the medial prefrontal cortex, cortical infralimbic area/Brodmann area 25, the diagonal band of Broca, midline para-ventricular thalamus, basal amygdala, subiculum and CA1 regions of the hippocampal formation, ventral tegmental area (VTA), nucleus tractus solitarius and dorsal raphe nucleus of the mesencephalon (Groenewegen et al., 1980, 1987, 1999; Burstein and Giesler, 1989; Berendse and Groenewegen, 1990; Berendse et al., 1992; Brog et al., 1993; Wright et al., 1996; Heimer et al., 1997; Perez-Santana et al., 1997; Delfs et al., 1998; Friedman et al., 2002; French and Totterdell, 2003; Van Dongen et al., 2005; Ikemoto, 2007; Thompson and Swanson, 2010).

The Göttingen minipig is increasingly replacing dogs and non-human primates in preclinical studies (Lind et al., 2007; Sauleau et al., 2009; Ganderup et al., 2012; Suenderhauf and Parrott, 2012). To adequately study novel neurosurgical interventional therapies in preclinical settings large animal models are needed as rodents' brains are too small and do not allow application of human DBS equipment (Bjarkam et al., 2009, 2016; Dolezalova et al., 2014). The Göttingen minipig has a large gyrated brain (6 × 5 × 4 cm) and is thus much more suitable for conventional MRI, PET imaging and translational studies involving neurosurgical techniques like cell replacement-based therapies and DBS than rodents (Bjarkam et al., 2004).

There are increasing ethical concerns when using non-human primates in experimental research. Accordingly, non-human primates should only be used as research animals when there are no *in vivo* or *in vitro* alternatives (Goodman and Check, 2002). The minipig provides such an *in vivo* alternative. Non-human primates are also difficult to procure, to keep and they require expensive housing facilities. In comparison pigs are affordable and straight forward to keep in regular animal housing. Pigs can thus represent an effective large animal model to be used in preclinical cell replacement modeling replacing non-human primates in the study of neurodegenerative disorders (Dolezalova et al., 2014).

In order to apply neurosurgical techniques to pigs in a preclinical setting it is important to study and understand the basic anatomical and physiological properties of the pig brain as variations between species occur.

The aim of this study was to investigate the retrograde connections of the NAcc in the Göttingen minipig by an MRI-guided stereotactic retrograde tracing-procedure, as well as to describe the basic anatomy of the NAcc in the Göttingen minipig by various histological and immunohistochemical procedures.

## Materials and methods

All experimental protocols were approved by the Danish Council of Animal Research Ethics (DANCARE). 6 Göttingen minipigs were used in the basic anatomy study of NAcc and 5 other animals successfully received an MRI-guided injection of 1 μL FluoroGold (FG a selectively fluorescent retrograde tracer, hydroxyl-stilbamidin methanesulfonate from Fluorochrome, Denver, CO; CAT no H22845, Molecular Probes Inc. diluted to 2% in distilled water) on one side of the brain in the tracing part.

**Figure 1:**
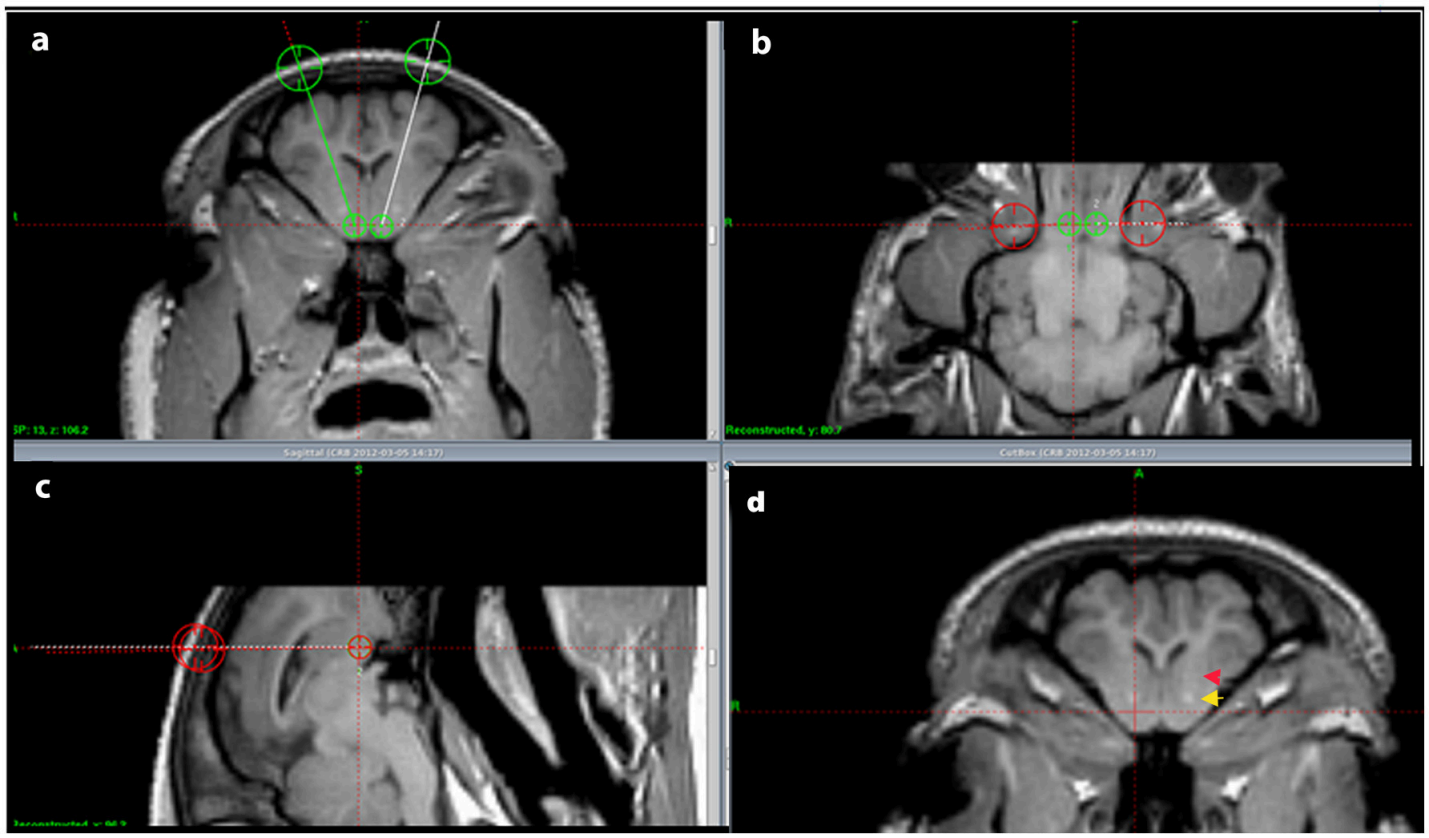
**Injection pathway. (A–C)** show the located NA and the intended injection pathways in a coronal **(A)**, horizontal **(B)**, and sagittal **(C)** cut view. In **(D)** the anterior commissure is marked by a yellow arrow and the internal capsule by a red arrow.

In the tracing part of the experiment the animals were sedated using an intra-muscular injection of 6 ml Midazolam (1 mg/ml) and 4 ml Ketamine (25 mg/ml). Intravenous access was obtained by ear vein catherization. To allow endotracheal intubation the animals received an intravenous injection of 3 ml Midazolam (1 mg/ml) and 2 ml Ketamine (25 mg/ml). Throughout the rest of the surgical procedure the animals were anesthetized by ventilation with Sevoflurane (1–2%). The pigs were then placed in an MRI compatible localizer box with applicable side-fiducials. The localizer box was fixed to the skull by pointed titanium screws in the os zygomaticus (Bjarkam et al., 2004). Next an MRI-scan was carried out in accordance with the protocol described in Bjarkam et al. (2009) and the images were transferred to a Leksell SurgiPlan system from which we could localize the NAcc and calculate the coordinates for the injection point and pathway of the tracer substances (Figure 1). Afterwards, the side-fiducials were replaced by a modified stereotaxic Leksell frame (Bjarkam et al., 2009) with an attached Hamilton micro syringe. A scalp incision was made followed by a drill hole using a Midas Rex power drill exposing the dura mater covered surface of the brain. The dura was gently cut open with a dura knife and the syringe stereotaxically guided into the NAcc target area. The injection of 1 μL was carried out in accordance with (Sørensen et al., 1995) resulting in a slow stepwise retraction of the syringe. The scalp incision was sutured in one layer and the animals made sure to breathe on their own prior to extubating.

The animals were afterwards kept, at the university large animal research facility, for 2 weeks in order to ensure retrograde axonal transportation of the tracer. At the day of sacrifice the tracing study animals and the basic anatomy study animals were first sedated and then euthanized by an overdose (20 ml) of 40% pentobarbital and transcardially perfused (Ettrup et al., 2011)with approximately 5 l of phosphate buffered 4% paraformaldehyde (pH 7.4). Afterwards the brains were removed and placed in formaldehyde for 1 week and then cut in 9 mm coronal brain slabs using a HistOtech brain slicer (Bjarkam et al., 2001). It was then immersed in a sucrose solution (30% phosphate buffer) for 1 week before freezing and final cryostat sectioning into 40 μm coronal sections.

One series was mounted with Depex without any further processing, another followed by Nissl-staining and a third by myelin staining using an autometallographic (AMG) technique (Larsen et al., 2003). Two series were kept free floating in a cryoprotective ethylene glycol solution (deOlmos) and immunohistochemically stained for DARPP-32 and calbindin.

The anti DARPP-32 (Dopamine and cAMP-regulated neuronal phosphoprotein) immunohistochemical procedures were performed in accordance with the avidin-biotin method (Bjarkam et al., 2004, 2005). Anti-DARPP-32 was applied as primary antibodies, whereas the secondary antibody was biotin-labeled anti-sheep IgG. The sections were rinsed in tris-buffered saline (TBS) + 1% Triton X-100 for 15 min and incubated with avidin 0.1% for 20 min, followed by another rinse with TBS for 2 min and incubation with biotin 0.01% for 20 min. After another 2 min TBS rinse the sections were pre-incubated with 1% TritonX-100 and 0.2% milk in TBS for 30 min. The primary antibodies were then added to these solutions and stored overnight at 4°C. Next day the sections were rinsed with TBS + Triton for 3 × 15 min and incubated with the secondary biotinylated antibody (1.400 in TBS 1% Triton + 0.2% milk) for 1 h. This was followed by a quick rinse in TBS + Triton and a blockade of endogenous tissue peroxidase with a solution consisting of TBS, methanol and hydrogen peroxide for 15 min. After another 3 × 15 min. TBS + 1% Triton rinse, the avidin peroxidase (diluted 1:400 in TBS + 1% Triton and 0.2% milk) was applied for 1 h at room temperature. Another 3 × 15 min TBS + 1% Triton rinse followed, and the avidin-peroxidase complex was visualized by incubation for 10 min with 0.1% diaminobenzidin (DAB) solution including 0.3% hydrogen peroxide. The sections were finally to be mounted with Depex.

Other brain sections were stained free-floating with monoclonal mouse Anti-Calbindin antibody (Abcam, ab82812) diluted 1:1000. Briefly, sections were washed in TBS, and the activity of the endogenous peroxidase was blocked by 3% H_2_O_2_ + 10% metanol in TBS. Target retrieval was performed by heating the sections for 30 min in DAKO retrieval buffer in 80°C followed by cooling of the sections for further 30 min. Then, sections were pre-incubated with 0.2% milk and incubated with primary antibody overnight in 4°C followed by incubation with biotinylated anti-mouse IgG diluted 1:200 in TBS + triton (RT) and finally for 1 h in ABC Vectastain kit for 1 h in RT. The DAB reaction was carried out for 5 min.

Myelin staining was carried out using an autometallographic (AMG) technique (Larsen et al., 2003). Sections on glass slides were placed in jars and covered with the AMG developer. The sections were allowed to develop for 1–2 h in a water bath at 26°C covered by a light-tight lid. Then the development was stopped by replacing the AMG developer with 5% thiosulphate for 10 min. The glass slides were then rinsed several times in distilled water. Counterstaining was performed with 0.1% toluidine blue in citrate buffer, pH 4.0. Finally the sections were rinsed twice in distilled water, dehydrated in 99% alcohol, imbibed with xylene, mounted with Depex, and coverslipped.

To study the cell sizes a number of randomly chosen cells were measured using ImageJ program on the microphotographs using 20x objective, taken from the five areas: the dorsal-lateral, the dorsal-medial, the middle, the ventro-lateral and the ventro-medial parts of NAcc (in total 500 cells were measured). Cell density was described qualitatively based on microscopic observations of the five microscopic coronal sections from different parts of the NAcc in the anterio-posterior axis.

The identification of fluorogold traced neurons was carried out through a thorough fluorescence-microscopic search for tracer substance in all the cut brain sections. The staining intensity and distribution within the various areas were allocated to either one of three descriptive categories: weak, medium and strong tracing. Identification and naming of traced structures and neighboring areas were carried out by reference to the Göttingen minipig brain atlas (Bjarkam et al., 2016).

## Results

### Anatomy and dichotomy

**Figure 2:**
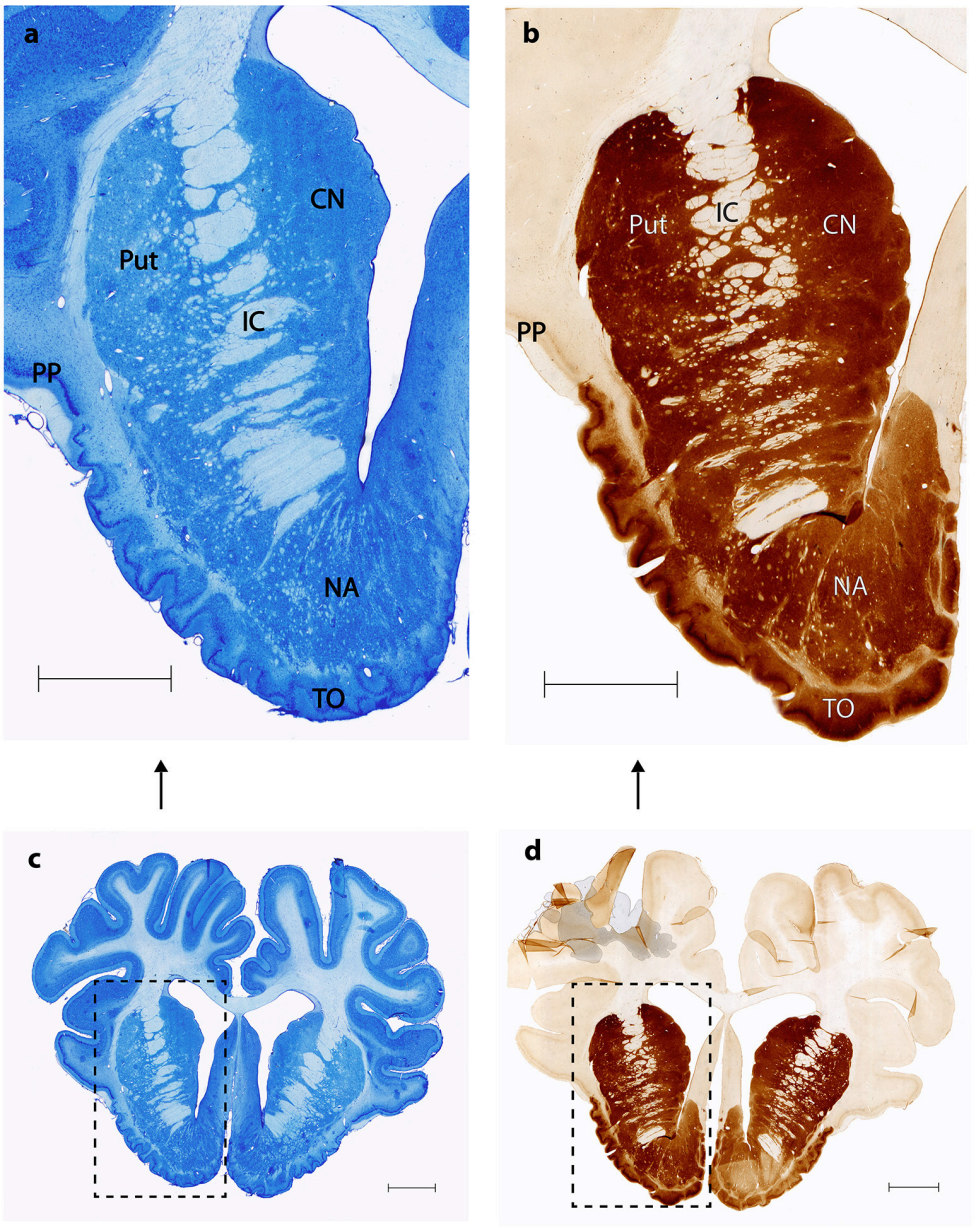
**Nucleus accumbens Nissl and anti DARPP-32 staining. (A,C)** show Nissl stained coronal sections of the nucleus accumbens (NA), the internal capsule (IC), the putamen (Put), the prepiriform cortex (PP) and the olfactory tubercle (TO). **(B,D)** Corresponding anti-DARPP-32 staining. Scale bar = 5 mm.

**Figure 3:**
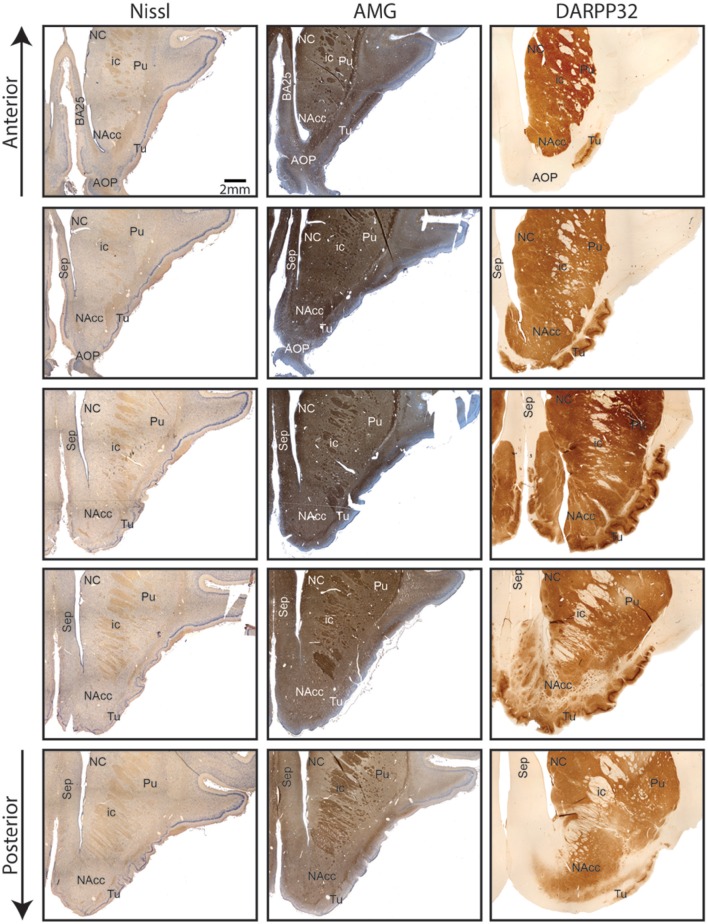
**Continuous coronal sections of nucleus accumbens stained with NISSL, autometallographic (AMG) myelin staining and anti-DARPP-32 staining showing nucleus accumbens (NAcc), the internal capsule, caudate nucleus (NC), putamen (Pu), the posterior part of the anterior olcaftory nucleus (AOP), olfactory tubercle (Tu), septum (Sep), and Broadmann Area 25 (BA25)**.

Nissl and anti DARPP-32 staining show how the anatomical and topographical relations of the Göttingen minipig NAcc are similar to that of humans (Voorn et al., 2004; Basar et al., 2010; Lucas-Neto et al., 2013). DARPP-32 is first and foremost localized to regions that receive dopaminergic innervation and the highest DARPP-32 levels are found in caudatoputamen, NAcc, olfactory tubercle, bed nucleus of stria terminalis, and portions of the amygdaloid complex with DARPP-32 immunoreactivity being present in neuronal cell bodies and dendrites (Hemmings et al., 1984; Svenningsson et al., 2004). Thus, anti DARPP-32 staining enables us to visualize the striatum including NAcc. As seen in Figures 2, 3 the minipig NAcc forms the main part of the ventral striatum where the head of the caudate nucleus and the ventroanterior part of the putamen meet beneath the anterior limb of the internal capsule, with AMG-myelin stained sections clearly visualizing the internal capsule. Ventrally NAcc is seen bordering the easily recognizable olfactory tubercle and medially the septum, subgenual cortex, infralimbic cortex, and the anterior horn of the lateral ventricle (Bjarkam et al., 2016). As in previous studies we have not been able to demonstrate any sharp borders between NAcc and the rest of the striatum in the caudal, lateral, and dorsal projections, but a dorsal border will by definition be set as ventral to the anterior commissure. The ventral border to the olfactory tubercle and the lateral border to prepiriform cortex can, likewise, be determined by assistance of the Nissl and anti-DARPP-32 stained sections (Figure 3).

The cerebral MRI scans allowed us to clearly identify the NAcc and confirm the basic anatomical findings above (Figure 1).

**Figure 4:**
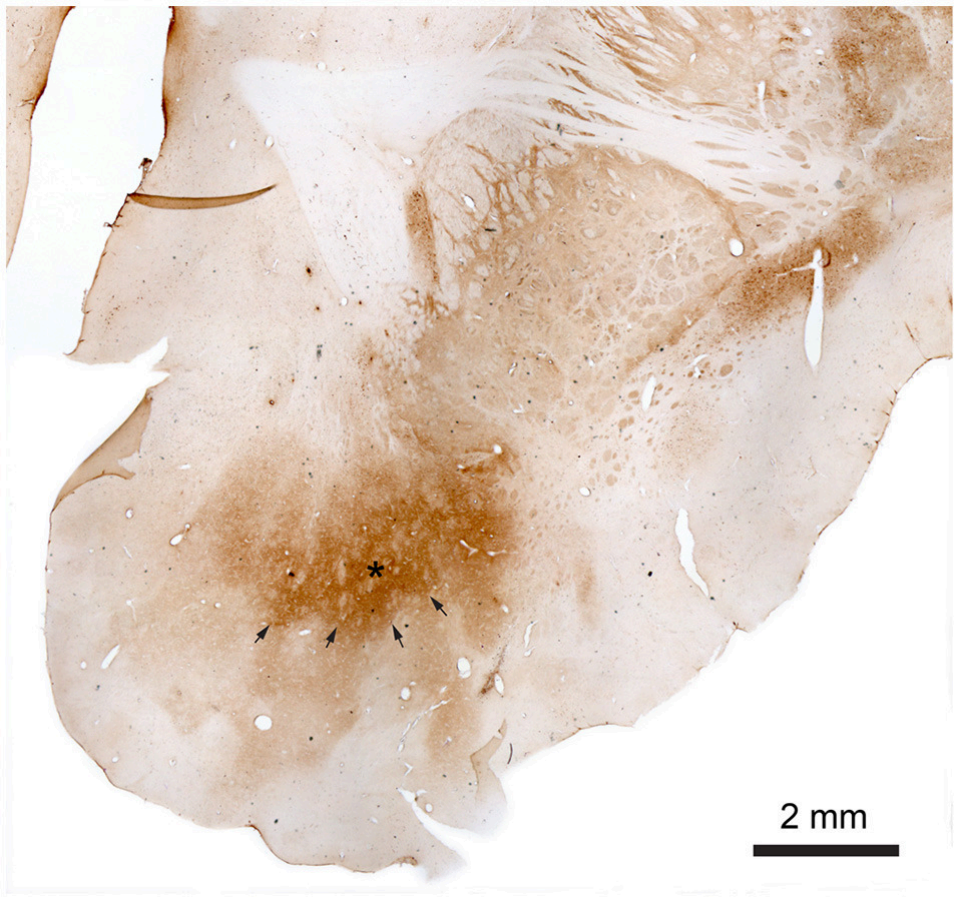
**Nucleus accumbens stained with monoclonal mouse Anti-Calbindin antibody**. The star marks the core of the NAcc and the arrows mark the contours of the core toward the shell. The shell more gradually fades out toward the border of the NAcc.

Antibody against Calbindin (CaB) was shown to stain cells and matrix within striatum (caudate, putamen, and less intensively globus pallidus) including NAcc (Figure 4). Fibers in the internal capsule remained unstained. CaB staining intensity within NAcc (in core) is strongest in its medio-rostral part and slightly lower in the caudal part. It seemed that most of the staining is a matrix staining, some CaB positive cell bodies could also be found; more positively stained cell bodies were seen in the caudal part of the NAcc.

The observed staining pattern is a bit similar to that seen in rats (Meredith et al., 1996). The difference in staining intensity within the NAcc gives rise to a very clear divisional dichotomy with a strongly stained core and weakly a stained shell. The border between the core and shell is quite visible, especially in the rostral part of NAcc. In this part of NAcc, the core is located dorsally, with the shell below it, and extends from the medial to the ventral part of the brain hemisphere. The core area is quite large with well-defined borders. The CaB staining within the core is uneven and within strongly stained areas some weakly stained patches could be found. Meanwhile the border between the NAcc core and shell is quite visible, the outer border of the shell is in some brain sections not well defined because of the low contrast between NAcc shell staining and background staining. Staining within the shell appears more homogeneous than in the core and gradually fades toward the outer border of the NAcc.

Toward the caudal part of the NAcc the shell starts surrounding the core. In the most caudal part NAcc consists of a small fragment close to the midline. It can, however, still be divided into two parts based on the staining intensity. The parts with stronger staining, lying on the dorsal and ventral part, and the middle part with weaker staining.

We found that the average soma diameter of neurons in the NAcc was 9.5 μm and no significant changes in diameter were observed in the anterior-posterior axis. Average diameter of the cell bodies measured in the dorsal part of the anterior NAcc was a bit smaller in the lateral parts than in the middle of the NAcc (8.89 ± 1.49 μm vs. 10.19 ± 1.38 μm, n.s.) and cell density in the lateral parts was found to be lower than in remaining parts of NAcc through qualitative assessment. In the posterior parts of the NAcc the difference in cell soma diameter between the lateral and medial parts disappears. However, we see a slight difference in the cell density between the dorsal and ventral part of the posterior NAcc (with lower cell density in the ventral part).

### Tracing

**Figure 5:**
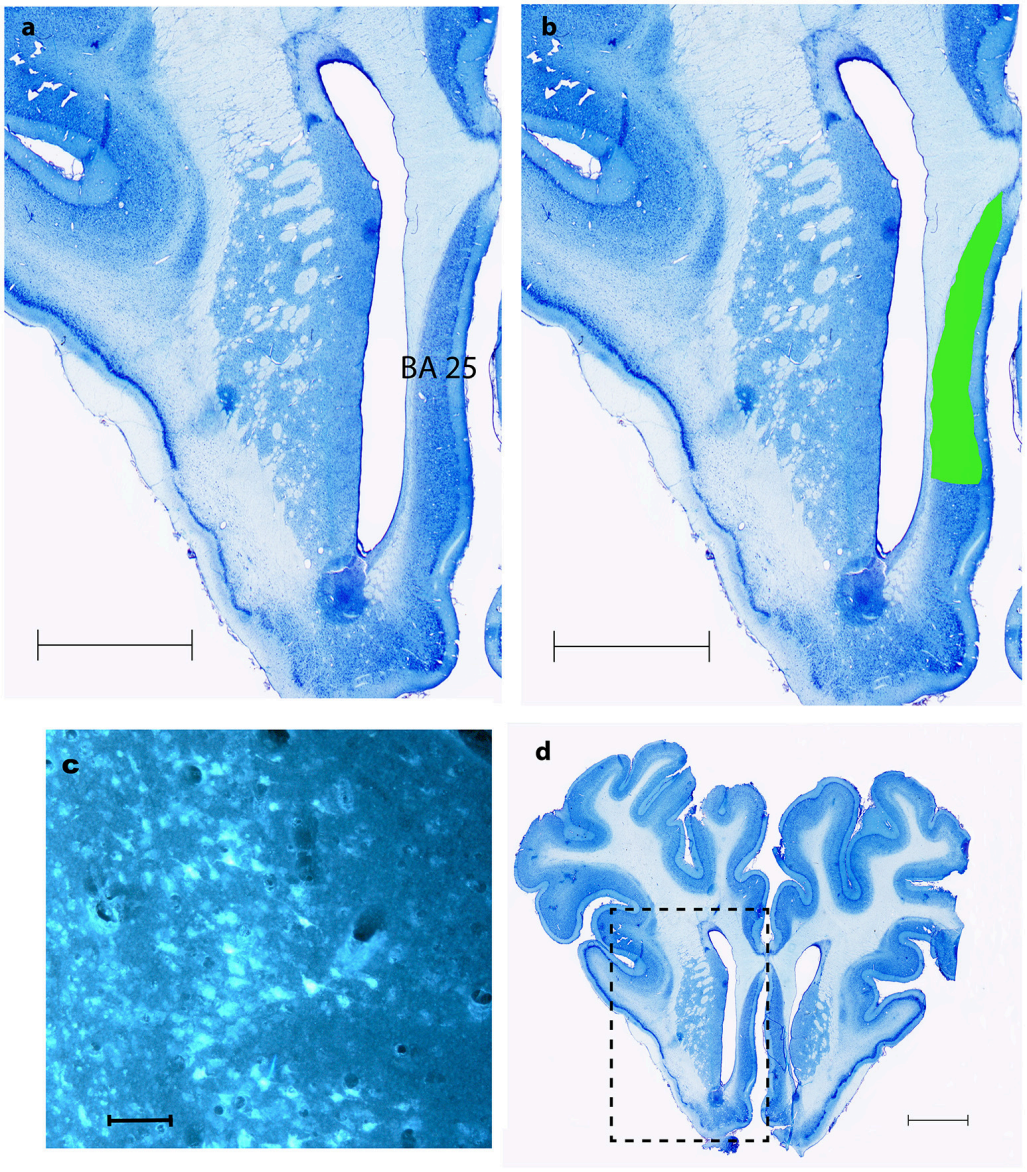
**BA 25. (A,B,D)** show coronal Nissl- staining with scale bars = 5 mm. BA 25 is marked with green in **(B,D)**. **(C)** Shows fluorescence microscopy of FG traced neurons in BA 25, scale bar = 200 μm.

**Figure 6:**
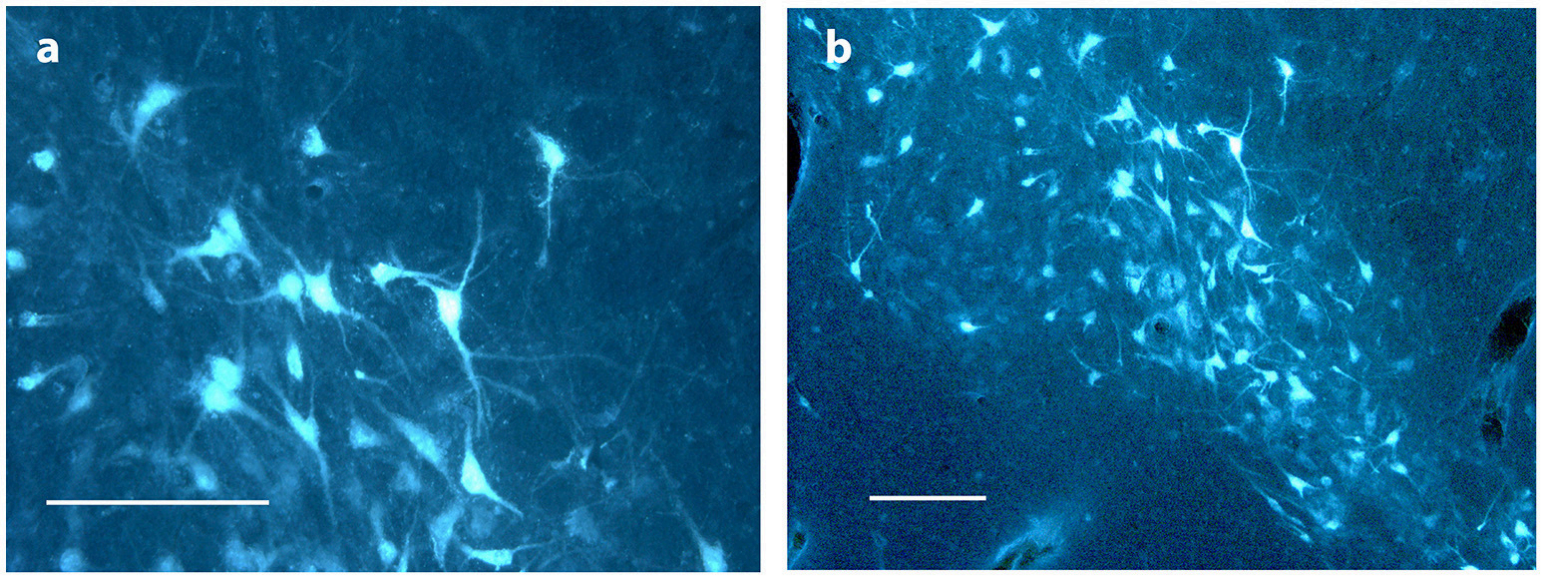
**Substantia nigra. (A,B)** show FG traced neurons in substantia nigra in Göttingen minipig ACNM 13. Scale bars = 200 μm.

As shown in Table 1 the FG tracing technique revealed inputs to NAcc originating from medial parts of the prefrontal cortex, BA 25 (subgenual cortex), the contralateral NAcc, basal amygdala, ventral CA1 region of hippocampus and entorhinal cortex, subiculum, the paraventricular and anterior parts of thalamus, dorsomedial parts of hypothalamus on the border between thalamus and hypothalamus, insula bilaterally, medial forebrain bundle (MFB), substantia nigra, VTA, retro rubral field (RRF), and dorsal and median raphe nuclei. FG was also found in fibers in the anterior commissure and the MFB. Figure 5 demonstrates FG-tracing to BA 25 and the medial prefrontal cortex and Figure 6 demonstrates FG-tracing to substantia nigra.

**Table 1:** **List of the anatomical structures where FG traced neurons were found**.

**Traced areas/animal number**	**1**	**2**	**3**	**4**	**5**
Prefrontal cortex medial		ttt	ttt+b	ttt+b	ttt
Area 25/subgenual cortex	ttt	ttt	ttt+b	ttt	ttt
Insula	ttt	ttt+b	tttb	ttt+b	tt
Prepiriform cortex		t		/	t
Perirhinal cortex	tt	t	/	tt	tt
Cingulate cortex	/	/	t	t	/
Septum	/	/	/	/	/
Anterior commissure	t+b	t+b			t+b
Hippocampus CAl	t	tt	ttt	t	t
Subiculum	/	/	/	/	/
Regio diagonalis		t			t
Amygdala	ttt	tt	ttt	ttt	ttt
Anterior olfactory nucleus		t		/	tt
Entorhinal cortex	t	t	tt	tt	tt
Nucleus habenularis	t			tt	
Thalamus paraventricular/medial	tt	tt	tt	ttt	ttt
Thalamus ventromedial		t	/		/
Thalamus, anterior			ttt	tt	ttt
Thalamus ventral				ttt	/
Border between thalamus and hypothalamus	t	t	tt	t	t
Raphe nucleus, dorsalis	tt+b	t+b	tt+b	t+b	t+b
Raphe nucleus median	t+b	t+b	t+b	t	t
MFB–medial forbrain bundle	ttt	tt	tt	ttt	ttt
VTA	ttt	ttt+b	tt+b	ttt+b	ttt+b
Substantia nigra	ttt+b	ttt+b	ttt+b	ttt+b	ttt+b
RRF (A8)			t	tt	tt

ttt, strong tracing; tt, medium tracing; t, weak tracing; b, bilateral. In the anterior commissure and MFB traced fibers and not neurons were seen.

In the first pig the injection was made in the caudal part of NAcc. In the second it was more rostral in the NAcc. The injection in third was more ventral, medial and caudal, whereas it was more ventral in the fourth. As for the fifth pig the injection was in the dorsal, caudal part of the NAcc.

In mesencephalon very heavy tracing was found bilaterally in neurons in substantia nigra (A9)—Figure 6—in all pigs, but most pronounced on the ipsilateral site of the injection. FG tracing was also quite pronounced in the ventral tegmental area VTA (A10) bilaterally and in the retro rubral field (A8) unilaterally. Traced neurons were also found bilaterally in both the median and dorsal raphe nucleus in all pigs. Fiber tracing in the MFB was found in all pigs.

In diencephalon intense FG tracing was found unilaterally in the paraventricular parts of thalamus. FG was found in the anterior thalamic structures in pig number 3, 4, and 5, but not in number 1 and 2. Traced neurons in the ventral thalamus were only seen in number 4 and not in any other brains. Tracing to the ventromedial thalamus was only seen in number 2. In hypothalamus the tracing was less pronounced, but weak to moderate neuronal tracing was found in the dorsomedial parts of hypothalamus on the border to thalamus in all pigs.

In telencephalon heavily labeled cells were consistently seen in the medial parts of the prefrontal cortex, most pronounced on the same site as the injection. Also prominent tracing to the insula was persistently seen in all the pigs. In three out of five animals the insular tracing was bilateral. The tracing was, however, much more moderate on the contra lateral site of the injection. Labeled neurons in the perirhinal cortex were seen in four pigs. Amygdala was also heavily FG labeled.

Consistent and strong labeling of cells was seen in Brodmann area 25. In one animal (number 3) more moderate contra lateral tracing was also noted.

In the ventral CA1 region of the hippocampus and subiculum weak, but evident, tracing was found in neurons in animal number 1, 2, 4, and 5. Whereas very strong tracing was found in the ventral CA1 region and subiculum in number 3. Weak but evident tracing in neurons was also found in gyrus cinguli in number 4 and 5. Moderate tracing was found in the anterior olfactory nucleus (AON) in number 2 and 5 and in the habenular nucleus in 1 and 4.

No tracing was found in any other parts of the striatum aside from the NAcc, nor in any other cortical areas than those mentioned above. Also, no tracing was found in the cerebellum. Septum was, likewise, carefully examined in all brains, but no tracing was seen.

Traced fibers were also found in the anterior commissure and in the MFB in all pigs.

## Discussion

The afferent connections to the NAcc in the Göttingen minipig are for most parts in agreement with the connections found in previous studies, primarily in rats. Inputs from structures such as VTA, substantia nigra, the paraventricular parts of thalamus, hypothalamus, medial parts of the prefrontal cortex, insula, the dorsal raphe nucleus, hippocampus, subiculum and amygdala were all found in both the studied literature and in this experiment (Groenewegen et al., 1980, 1987, 1999; Burstein and Giesler, 1989; Berendse and Groenewegen, 1990; Berendse et al., 1992; Brog et al., 1993; Wright et al., 1996; Heimer et al., 1997; Perez-Santana et al., 1997; Delfs et al., 1998; Friedman et al., 2002; Van Dongen et al., 2005; Ikemoto, 2007).

To our knowledge only one other study (Haber et al., 1995) found tracing to the BA 25. This is very interesting considering, that this is another target for DBS treatment of TRD and OCD in humans, and because BA 25 is thought to play an important role in the pathophysiology of the former condition (Hauptman et al., 2008). The therapeutic effects of NAcc-DBS and BA25-DBS might accordingly be elicited by stimulation of a limbic circuitry anchored in these two structures. Descriptions of projections from the AON, the cingulate cortex and nucleus habenularis to the NAcc were not to our knowledge shown in previous rat or cat studies. Tracing to the AON was only found in ACNM 2 and 5. Considering how close the AON is located to the NAcc it seems likely to be spill-over tracing from the NAcc injection site. Hence this study does not provide sufficient evidence to conclude the existence of connections to the NAcc originating from AON. Traced neurons in the cingulate cortex were only found in animals 3 and 4. The injection in these pigs was made in the ventral part of NAcc, and it is possible that the ventral NAcc receives different afferent connections from the rest of the NAcc. However, further anterograde studies from the cingulate cortex in the Göttingen minipig are needed to verify if such connections exist. Traced fibers in the anterior commissure show that this very likely is the way by which the NAcc receives its afferents from the contra lateral side of the brain. We cannot, however, rule out that some amount of tracer substance might have spread to the anterior commissure. The injections in pigs, number 1, 2, and 5 were, however, in very different areas of the NAcc, why it seems most likely to be significant.

Tracer spread into neighboring areas of the injection site can never entirely be ruled out despite the use of a very precise MRI-guided stereotaxic injection method. The consistency of the results in all five pigs with injection sites found in various locations of the nucleus, as well as, the fact that the findings align with previous studies in non-human primates and rodents, however, makes our findings credible.

Heimer et al. described how in primates various projections to the NAcc are topographically organized and that various brain areas predominantly seem to project to certain parts of the NAcc (Heimer et al., 1997). Even though our findings are in agreement with that of Heimer our study calls for future topographical projection studies in Göttingen minipig as DBS or cell replacement therapies could have different effects depending on what area of NAcc that is targeted if there same topographical pattern were to be found in the minipig as in primates. However, such studies would currently only have post-mortem implications in neuro interventional studies with DBS and cell replacement therapies in the sense that current MRI scanners and stereotactic devices as seen in Figure 1 would not enable one to certify more precisely where for example DBS electrodes had been placed within the NAcc due to its quite complicated complicated neurochemical anatomy and the precision of stereotactic devices and MRI resolutions.

This study supports the role of the NAcc found in previous studies in different species and indicates how the Göttingen minipig NAcc with numerous direct projections from limbic areas most likely is involved in the processing of emotional reactions (amygdala), information of the state of arousal (midline brain stem and thalamus) and contextual information via inputs from the hippocampus and the entorhinal cortex. Also the medial prefrontal connections signify how the NA likely is involved in the processing of cognitive information and executive functions. The strong connections from the substantia nigra, VTA and RRF add verification to the role of NAcc in motivational behavior, whereas the projections from the raphe nuclei suggest a role in mood regulation and behavior and a potential involvement in the pathogenesis of severe depression. The study also showed strong connections to NAcc from BA 25 and might explain some of the therapeutic effects of DBS against TRD in NAcc.

This study limited its focus to the investigation of the retrograde connections, segregation, and dichotomy of NAcc. Future studies of the anterograde connections and more detailed descriptions of the cytology of the Göttingen minipig are much called for.

In conclusion the Göttingen minipig NAcc can be clearly divided into a core and shell and is a highly developed neural structure with prominent retrograde connections from several subrhinal and pre/infra limbic targets, which could make it a promising experimental model for DBS studies of TRD and OCD.

## Role of authors

All authors had full access to all the data in the study and take responsibility for the integrity of the data and the accuracy of the data analysis.

## Author contributions

AM: Principal investigator and writer of the manuscript. Carried out most of the experiments and performed the data analysis and interpretation. Contributed to the study concept and design. DO: Was essential in dividing the NAcc into a core and shell. Undertook critical revision of the manuscript for important intellectual content. JS: Study concept and design. Obtained funding. Critical revision of the manuscript for important intellectual content. CB: Study concept and design. Undertook critical revision of the manuscript for important intellectual content.

### Conflict of interest statement

The authors declare that the research was conducted in the absence of any commercial or financial relationships that could be construed as a potential conflict of interest.
